# Predictive performance of the Modified Frailty Index and ASA-PS classification for 30-day mortality after esophagectomy in patients with esophageal cancer

**DOI:** 10.3389/fonc.2026.1818115

**Published:** 2026-07-22

**Authors:** Wongsakorn Chaochankit, Somkiat Sunpaweravong, Chutida Sungworawongpana

**Affiliations:** 1 Division of General Surgery, Department of Surgery, Faculty of Medicine, Prince of Songkla University, Songkhla, Thailand; 2 Department of Anesthesiology, Faculty of Medicine, Prince of Songkla University, Songkhla, Thailand

**Keywords:** ASA physical status classification, esophageal cancer, esophagectomy, Modified Frailty Index (mFI-5), risk prediction

## Abstract

**Background:**

Esophageal cancer is commonly diagnosed at a locoregional stage and is typically treated with neoadjuvant chemoradiotherapy followed by esophagectomy. Despite advances in perioperative care, esophagectomy remains associated with substantial morbidity and mortality. Frailty assessment has been proposed as a tool for preoperative risk stratification, but its predictive value in patients undergoing esophagectomy remains uncertain.

**Methods:**

We conducted a retrospective cohort study of patients who underwent curative-intent esophagectomy between January 2012 and December 2024. The predictive performance of the 5-item Modified Frailty Index (mFI-5) was compared with the American Society of Anesthesiologists Physical Status (ASA-PS) classification. Receiver operating characteristic (ROC) curve analysis and exploratory multivariable logistic regression were performed to evaluate the predictive performance of mFI-5 and ASA-PS classification for 30-day mortality.

**Results:**

A total of 123 patients underwent esophagectomy, with a 30-day mortality rate of 9.8%. Patients who died had significantly longer intensive care unit and hospital stays and a higher incidence of severe postoperative complications. ASA-PS demonstrated better discrimination than mFI-5 for predicting 30-day mortality (AUC 0.67 vs. 0.55), although the discriminatory performance of both tools remained limited. In the exploratory multivariable analysis, ASA-PS classification was independently associated with 30-day mortality, whereas mFI-5 was not.

**Conclusions:**

Although ASA-PS classification was independently associated with 30-day mortality, both ASA-PS and mFI-5 demonstrated limited predictive performance following esophagectomy. These findings suggest that mFI-5 and ASA-PS alone provide limited discrimination for predicting 30-day mortality and support the development of esophagectomy-specific risk prediction models incorporating objective clinical, physiological, functional, and procedure-specific variables.

## Background

Esophageal cancer is a major global health concern, particularly in low- and middle-income countries. It ranks as the seventh most common cancer worldwide and the sixth leading cause of cancer-related death globally (1). In Asia, squamous cell carcinoma (SCC) accounts for about 80 percent of all esophageal cancers (2). Most patients diagnosed with esophageal cancer present at the locoregional stage (3), at which point the standard treatment involves neoadjuvant chemoradiotherapy followed by esophagectomy. While chemoradiotherapy has significantly improved in efficacy over the years, esophagectomy remains associated with high morbidity and mortality. Studies from high-volume centers over the last two decades have reported postoperative mortality rates of less than 5% (4). Despite these improvements, morbidity after esophagectomy remains a substantial challenge, with complication rates exceeding 60% in some series (5). The overall incidence of post-esophagectomy complications ranges from 20-68% (6). Several preoperative factors have been identified to increase the risk of complications and mortality following esophagectomy. These include advanced age, pulmonary compromise, malnutrition, and renal or hepatic dysfunction (6). Comorbidities such as cardiovascular disease, diabetes, chronic obstructive pulmonary disease (COPD), and hypertension further elevate the risk of postoperative complications like anastomotic leaks, cardiorespiratory issues, reoperation, and death (7).

In recent years, as the aging population continues to grow, there has been increasing attention on frailty as a predictor of adverse outcomes, especially in surgical settings. In community-dwelling adults aged 65-90, frailty prevalence is typical below 30% (8). A 2019 multicenter prospective cohort study on adult emergency surgical admissions found that worsening frailty, regardless of age, is strongly associated with poorer patient outcomes, including longer hospital stay, higher 30-day readmission and increased 30-day mortality (9). In the context of esophageal cancer, a study by Tang et al. evaluated the Esophagectomy Vitality Index and found it to be a more accurate predictor of post-esophagectomy morbidity and mortality than the widely used 11-factor Modified Frailty Index (mFI) (10). Despite this, the study’s small sample size (77 patients) and low incidence of the outcomes (1 mortality and 23% complication rate) suggest that further research is needed to better understand the full potential of frailty as a predictor in esophageal cancer surgery. This has sparked additional interest in the applicability of mFI as a frailty tool in this specific population.

The American Society of Anesthesiologists-Physical Status (ASA-PS) classification system is a grading system used to assess a patient’s fitness for surgery (11). While the ASA-PS provides an initial overview of a patient’s pre-anesthesia health and medical comorbidities, it has been criticized for its subjectivity and variability in inter-rater reliability (12). Despite this, there is increasing evidence that ASA-PS can also serve as a useful tool for predicting surgical morbidity and mortality. In fact, several recent studies suggest that poor ASA-PS scores are associated with poorer long-term prognosis in patients with esophageal cancer (13). However, the role of ASA-PS in predicting post-treatment outcomes for esophageal cancer patients remains an area of ongoing research and is not yet fully understood.

The preoperative identification of factors associated with post-esophagectomy morbidity and mortality offers an opportunity for patient optimization to minimize adverse outcomes. Although various frailty measures have been studied, there is currently no consensus on the most reliable method to identify patients with poor physiological reserve who are undergoing esophagectomy. Based on existing evidence, we aimed to compare the predictive performance of the mFI-5 and the ASA-PS classification for postoperative complications and 30-day mortality following esophagectomy for esophageal cancer. We hypothesized that these commonly used preoperative assessment tools would demonstrate different predictive performance in this patient population.

## Materials and methods

### Study design and setting

This retrospective cohort study was conducted at Songklanagarind Hospital, a tertiary care academic hospital in Thailand. The study reviewed medical records of consecutive patients diagnosed with esophageal cancer who underwent esophagectomy between January 2012 and December 2024. Ethical approval was obtained from the Human Research Ethics Committee (HREC) of the Faculty of Medicine, Prince of Songkla University. The study adhered to the Strengthening the Reporting of Observational Studies in Epidemiology (STROBE) guidelines to ensure transparency and rigor in reporting.

### Study population

The study included adult patients with histologically confirmed esophageal squamous cell carcinoma or adenocarcinoma who underwent curative-intent esophagectomy and had complete data for the variables required for analysis. Patients younger than 18 years of age, those who underwent palliative esophagectomy, those with synchronous malignancies, or those with distant metastatic disease at the time of surgery were excluded.

### Data collection

Data were extracted from electronic medical records and institutional databases using a standardized data collection form. Collected variables included demographic characteristics (age, sex, body mass index [BMI], smoking history, and alcohol history). Smoking and alcohol exposure were recorded as binary variables (ever vs. never). Preoperative clinical characteristics included ASA-PS classification, comorbidities, mFI-5, pulmonary function, tumor stage, histological subtype, and receipt of neoadjuvant or adjuvant chemoradiotherapy. Intraoperative variables included the type of esophagectomy, minimally invasive approach, operative time, estimated blood loss, and epidural anesthesia. Postoperative outcomes included ICU length of stay, hospital length of stay, Clavien–Dindo classification, and 30-day mortality.

### Frailty and risk stratification measures

Frailty was assessed using the mFI-5, which assigns one point for each of the following conditions: diabetes mellitus, congestive heart failure, COPD or recent pneumonia, dependent functional status at surgery, and hypertension requiring medication. Patients with an mFI-5 score of ≥2 were classified as frail, as this cutoff has been associated with an increased risk of postoperative adverse outcomes in previous studies. Two blinded investigators independently assessed frailty, and inter-rater reliability was measured using Cohen’s kappa coefficient. The ASA-PS classification was recorded from preoperative anesthesia assessments and categorized into ASA-PS I-II, indicating lower perioperative risk, and ASA-PS III-IV, indicating higher perioperative risk.

### Study outcomes

The primary outcome was 30-day postoperative mortality, defined as death occurring within 30 days after esophagectomy. Secondary outcomes included postoperative complications classified according to the Clavien–Dindo classification.

### Statistical analysis

Descriptive statistics were performed, with continuous variables presented as mean (standard deviation [SD]) or median (interquartile range [IQR]), depending on data distribution, and categorical variables summarized as frequencies and percentages. Continuous variables were compared using the Student’s *t*-test or Wilcoxon rank-sum test, according to data distribution, whereas categorical variables were compared using the Pearson chi-square test or Fisher’s exact test.

Univariable logistic regression analysis was initially performed to evaluate the association between baseline variables and 30-day mortality. An exploratory multivariable logistic regression model was subsequently constructed to assess the independent association of three clinically relevant preoperative variables selected *a priori* (mFI-5, ASA-PS classification, and smoking) with 30-day mortality. Because only 12 mortality events occurred, the multivariable model was intentionally restricted to these three variables to reduce the risk of overfitting. Adjusted odds ratios (ORs) with 95% confidence intervals (CIs) were reported. Receiver operating characteristic (ROC) curve analysis was performed separately for 30-day mortality and postoperative complications classified according to the Clavien–Dindo classification to evaluate the discriminatory performance of mFI-5 and ASA-PS classification. The area under the ROC curve (AUC), sensitivity, specificity, and accuracy were calculated.

Statistical analyses were performed using R statistical software (version 4.0.5) and IBM SPSS Statistics version 26.0 (IBM Corp., Armonk, NY, USA). All statistical tests were two-sided, and a *p* value <0.05 was considered statistically significant.

## Results

This study included 123 patients with esophageal cancer who underwent esophagectomy. Among them, 12 patients (9.76%) died within 30 days after surgery, while 111 patients (90.24%) survived.

### Demographic and baseline characteristics

Baseline demographic and clinical characteristics are summarized in **Table 1**. Most baseline characteristics were comparable between survivors and non-survivors. Age, sex, smoking status, alcohol consumption, diabetes mellitus, hypertension, functional status, mFI-5 score, tumor stage, and receipt of chemoradiotherapy did not differ significantly between the two groups. COPD, squamous cell carcinoma, and lower preoperative FEV1 tended to be more frequent among non-survivors, although these differences did not reach statistical significance.

**Table 1:** Demographic characteristics of esophageal cancer patients who underwent esophagectomy (N = 123).

Variables	Death (n=12, 9.76%)	Alive (n=111, 90.24%)	P value
Age (years, IQR)	65.5 (60.8, 68.5)	62 (56, 66)	0.234
Male (n, %)	11 (91.7)	102 (91.9)	1.000
Smoking (n, %)	11 (91.7)	84 (75.7)	0.294
Alcohol consumption (n, %)	5 (41.7)	64 (57.7)	0.451
Diabetes mellitus (n, %)	0 (0)	9 (8.1)	0.597
COPD (n, %)	4 (33.3)	15 (13.5)	0.090
Dependent functional status (n, %)	0 (0)	1 (0.9)	1.000
Hypertension (n, %)	2 (16.7)	18 (16.2)	1.000
5-point mFI score (n, %)			0.441
0	8 (66.7)	77 (69.4)	
1	2 (16.7)	26 (23.4)	
2	2 (16.7)	7 (6.3)	
3	0 (0)	1 (0.9)	
Stage (n, %)			0.203
I	0 (0)	5 (4.5)	
II	5 (41.7)	39 (35.1)	
III	5 (41.7)	63 (56.8)	
IV	2 (16.7)	4 (3.6)	
Squamous cell carcinoma (n, %)	12 (100)	84 (75.7)	0.067
Preoperative CRT (n, %)	11 (91.7)	87 (78.4)	0.456
Postoperative CRT (n, %)	0 (0)	13 (11.7)	0.360
FEV1 (mean, SD)	1.9 (0.4)	2.4 (0.5)	0.058
Ejection fraction (mean, SD)	70.5 (6.3)	68.1 (7.9)	0.497

All data are presented as median (IQR) unless indicated otherwise.

### Operative characteristics and postoperative outcomes

Operative details and postoperative outcomes are presented in **Table 2**. Patients who died within 30 days were more likely to have a higher ASA-PS classification (p = 0.027). Operative approach, minimally invasive surgery, cervical anastomosis, operative time, estimated blood loss, and epidural anesthesia did not differ significantly between groups. Non-survivors had significantly longer ICU stay and hospital length of stay and were more likely to develop severe postoperative complications (Clavien-Dindo grade >2). The overall distribution of postoperative complications according to the Clavien–Dindo classification is presented in **Supplementary Table 1**. Overall, 60 patients (48.8%) experienced major postoperative complications (Clavien–Dindo grade >2), whereas 63 patients (51.2%) had grade 1–2 complications.

**Table 2:** Operative details and outcomes of esophageal cancer patients who underwent esophagectomy (N = 123).

Variables	Death (n=12, 9.74%)	Alive (n=111, 90.26%)	P value
ASA classification (n, %)			0.027
1	0 (0)	1 (0.9)	
2	4 (33.3)	77 (69.4)	
3	7 (58.3)	32 (28.8)	
4	1 (8.3)	1 (0.9)	
Operation (n, %)			0.724
Mc-Keown	2 (16.7)	21 (18.9)	
Ivor-Lewis	0 (0)	11 (9.9)	
Trans-hiatal	1 (8.3)	13 (11.7)	
Thoracoscopic and laparotomy	2 (16.7)	27 (24.3)	
Thoracolaparoscopic	6 (50)	34 (30.6)	
Thoracotomy and laparoscopic	1 (8.3)	5 (4.5)	
Minimally invasive surgery (n, %)	9 (75)	66 (59.5)	0.364
Cervical anastomosis (n, %)	9 (75)	100 (90.1)	0.139
Operative time (min, mean (SD))	572.5 (120)	528.8 (147.9)	0.325
EBL (mL, median (IQR))	225 (150, 312.5)	250 (150, 450)	0.738
Epidural anesthesia (n, %)	2 (16.7)	48 (43.2)	0.120
Retained intubation (n, %)	12 (100)	103 (92.8)	1.000
Reintubation (n, %)	0 (0)	2 (1.8)	1.000
ICU stay (days, median (IQR))	19.5 (9.5, 29.2)	3 (2, 4.5)	< 0.001
LOS (days, median (IQR))	29 (20, 37)	17 (13.5, 26)	0.035
Clavien-Dindo grade > 2 (n, %)	11 (91.7)	49 (44.1)	0.005

All data are presented as median (IQR) unless indicated otherwise.

**Figure 1:**
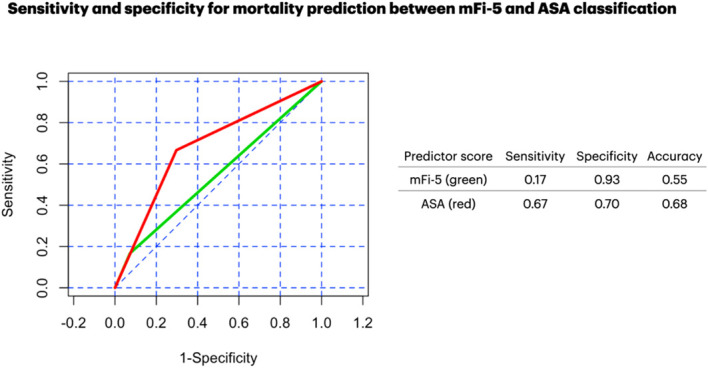
ROC curves for predicting 30-day mortality using the ASA-PS classification and the mFI-5. The AUC was 0.68 (95% CI 0.53-0.82) for ASA-PS classification and 0.55 (95% CI 0.45–0.68) for mFI-5.

**Figure 2:**
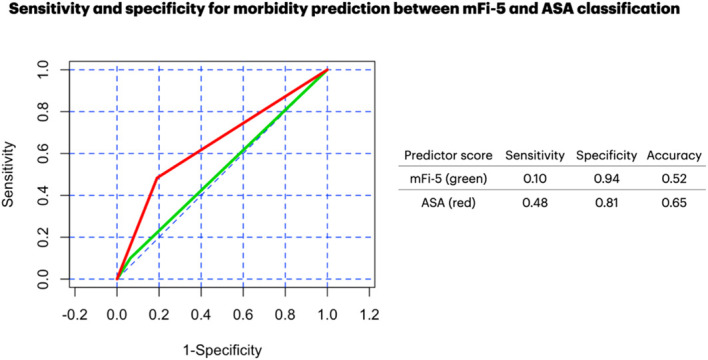
ROC curves for predicting major postoperative complications (Clavien–Dindo grade >II) using the ASA-PS classification and the mFI-5. The AUC was 0.65 (95% CI, 0.56–0.73) for ASA-PS classification and 0.52 (95% CI, 0.47–0.57) for mFI-5.

ROC analysis demonstrated limited discriminatory performance for both mFI-5 and ASA-PS classification in predicting 30-day mortality (**Figure 1**). The ASA-PS classification showed better discrimination than mFI-5 (AUC 0.67 vs. 0.55), whereas mFI-5 demonstrated higher specificity. Similar findings were observed for postoperative complications classified according to the Clavien–Dindo classification. ROC analysis for postoperative complications likewise demonstrated only modest discrimination for both assessment tools, with AUCs of 0.65 for ASA-PS classification and 0.52 for mFI-5 (**Figure 2**).

### Exploratory multivariable analysis

An exploratory multivariable logistic regression model including three clinically relevant preoperative variables (mFI-5 ≥2, ASA-PS classification ≥III, and smoking) was performed to evaluate factors associated with 30-day mortality (**Table 3**). Because only 12 mortality events occurred, the model was intentionally restricted to reduce the risk of overfitting.

**Table 3:** Exploratory multivariable analysis of factors associated with 30-day mortality of esophageal cancer patients (N = 123).

Variables	Adjusted odds ratio (95% CI)	P value
Modified frailty index ≥ 2	1.93 (0.33, 11.22)	0.484
ASA classification ≥ class 3	4.38 (1.21, 15.86)	0.020
Smoking	3.5 (0.41, 29.67)	0.185

The ASA-PS classification ≥III remained independently associated with increased 30-day mortality (adjusted OR 4.38, 95% CI 1.21–15.86; p = 0.020). Although mFI-5 ≥2 (adjusted OR 1.93, 95% CI 0.33–11.22; p = 0.484) and smoking (adjusted OR 3.50, 95% CI 0.41–29.67; p = 0.185) were associated with higher odds of mortality, neither reached statistical significance.

## Discussion

This study examined the predictive value of the mFI-5 and the ASA-PS classification for 30-day mortality following esophagectomy for esophageal cancer. Although ASA-PS classification demonstrated a significant association with 30-day mortality in the exploratory multivariable analysis, both ASA-PS and mFI-5 demonstrated only limited discriminatory performance for mortality prediction. These findings suggest that mFI-5 and ASA-PS provide limited prognostic information for predicting 30-day mortality after esophagectomy and highlight the need for more comprehensive perioperative risk prediction models. Our observed postoperative mortality rate of 9.76% is consistent with previously reported mortality rates of approximately 5%–10% in high-volume esophageal surgery centers (1, 2). Consistent with previous reports, ASA-PS demonstrated better discrimination than mFI-5 (AUC 0.67 vs. 0.55). However, the overall predictive performance of both assessment tools remained limited, suggesting that conventional frailty and ASA-PS assessments alone may not adequately capture the complexity of perioperative risk among patients undergoing esophagectomy (3, 4).

Frailty assessment has gained increasing attention in surgical oncology, with the mFI-5 commonly used to predict postoperative morbidity and mortality (8). However, in the present study, mFI-5 was not independently associated with 30-day mortality (adjusted OR 1.93, 95% CI 0.33–11.22; *p* = 0.484). This finding should be interpreted with caution because only 12 mortality events occurred, limiting statistical power and the precision of effect estimates. In addition, the mFI-5 primarily reflects baseline frailty and may not adequately capture procedure-specific risk factors unique to esophagectomy, such as surgical complexity, cardiopulmonary reserve, and perioperative physiological stress (9).

ASA-PS classification remains one of the most widely used tools for preoperative risk assessment, although its subjective nature and inter-rater variability are well recognized (11). In the present study, ASA-PS classification remained independently associated with 30-day mortality in the exploratory multivariable analysis. Nevertheless, its discriminatory ability remained limited (AUC 0.67), indicating that ASA-PS alone is insufficient for accurate risk prediction. These findings suggest that, although ASA-PS reflects baseline physiological status, it does not fully account for procedure-specific factors such as tumor characteristics, cardiopulmonary reserve, and the physiological demands of esophagectomy (13). One possible explanation for the limited predictive performance observed in this study is that both mFI-5 and ASA-PS are generic preoperative assessment tools rather than procedure-specific risk prediction models. The mFI-5 primarily reflects comorbidity-related frailty and does not incorporate functional performance, nutritional status, cognitive impairment, or physiologic reserve. Likewise, although ASA-PS summarizes overall physical status, its subjective nature and limited discriminatory range may reduce its ability to distinguish patients with different levels of perioperative risk. Furthermore, frailty measures may be more useful for predicting postoperative complications than early mortality alone, particularly in patients undergoing complex oncologic procedures such as esophagectomy. Preoperative pulmonary function (FEV1) also demonstrated a trend toward an association with 30-day mortality (p = 0.058). Although this association did not reach statistical significance, it is clinically plausible because impaired pulmonary reserve has consistently been associated with an increased risk of postoperative pulmonary complications and mortality following esophagectomy. The lack of statistical significance in the present study may reflect the limited sample size and the small number of mortality events.

Similarly, although smoking was associated with increased odds of 30-day mortality, the association did not remain statistically significant after multivariable adjustment. Because smoking exposure was recorded as a binary lifetime variable (ever vs. never), information regarding smoking intensity or pack-years was unavailable, which may have reduced its discriminatory ability. Nevertheless, cigarette smoking remains a well-established risk factor for adverse postoperative outcomes following esophagectomy through impaired pulmonary function, delayed wound healing, and increased postoperative pulmonary complications (14, 15). These findings support current recommendations for preoperative smoking cessation, although larger prospective studies are required to clarify its independent contribution to postoperative mortality.

The present findings suggest that mFI-5 and ASA-PS classification alone may not adequately reflect the complex physiological demands and procedure-specific risks associated with esophagectomy. Future efforts should focus on developing esophagectomy-specific prediction models that integrate objective clinical, physiological, functional, and procedure-specific variables to improve perioperative risk stratification (16, 17). Such models may provide more accurate individualized risk estimates than conventional frailty or ASA-PS assessment alone. Furthermore, optimized perioperative management strategies, including enhanced recovery pathways and individualized multidisciplinary care, may improve outcomes in patients undergoing esophagectomy (15, 18, 19).

This study has several limitations. First, the retrospective design may have resulted in missing data, information bias, and residual confounding because some clinically relevant variables were not routinely available in the medical records. Second, the relatively small sample size and the occurrence of only 12 mortality events limited statistical power and required restriction of the exploratory multivariable model to reduce the risk of overfitting. Third, smoking and alcohol exposure were recorded as binary lifetime variables (ever vs. never), precluding assessment of dose–response relationships or the effects of exposure intensity. Fourth, the inclusion of multiple operative techniques may have introduced clinical heterogeneity that could have influenced both postoperative morbidity and mortality, as different operative approaches are associated with varying operative complexity and perioperative risk profiles. Finally, this was a single-center study, which may limit the generalizability of the findings. In addition, the use of 30-day mortality may not capture deaths occurring between 30 and 90 days after esophagectomy, and the study population consisted of selected surgical candidates, which may have limited variability in frailty measures. Future prospective, multicenter studies with larger patient cohorts are warranted to validate these findings and to develop more robust esophagectomy-specific risk prediction models (17).

## Conclusion

These findings suggest that mFI-5 and ASA-PS alone provide limited discrimination for predicting 30-day mortality following esophagectomy. Future studies should focus on developing and validating esophagectomy-specific risk prediction models that integrate objective clinical, physiological, functional, and procedure-specific variables.

## Data Availability

The original contributions presented in the study are included in the article/**Supplementary Material**. Further inquiries can be directed to the corresponding author.

## Supplementary material

Supplementary Table 1 Distribution of postoperative complications according to the Clavien–Dindo classification.

## References

[1] Torre LA Siegel RL Ward EM Jemal A . Global cancer incidence and mortality rates and trends--an update. Cancer Epidemiol Biomarkers Prev. (2016) 25:16–27. doi: 10.1158/1055-9965.epi-15-0578 26667886

[2] Technology Ia . Hospital based cancer registry annual report 2014. National Cancer Institute (2016). Bangkok: Pornsup Printing Co. LTD.

[3] Batra R Malhotra GK Singh S Are C . Managing squamous cell esophageal cancer. Surg Clin N Am. (2019) 99:529–41. doi: 10.1016/j.suc.2019.02.006 31047040

[4] Orringer MB Marshall B Chang AC Lee J Pickens A Lau CL . Two thousand transhiatal esophagectomies: changing trends, lessons learned. Ann Surg. (2007) 246:363–72. doi: 10.1097/SLA.0b013e31814697f2 PMC1959358 17717440

[5] Naffouje SA Salloum RH Khalaf Z Salti GI . Outcomes of open versus minimally invasive Ivor-Lewis esophagectomy for cancer: a propensity-score matched analysis of NSQIP database. Ann Surg Oncol. (2019) 26:2001–10. doi: 10.1245/s10434-019-07319-6 30927192

[6] Kelly RJ Ajani JA Kuzdzal J Zander T Van Cutsem E Piessen G . Adjuvant nivolumab in resected esophageal or gastroesophageal junction cancer. N Engl J Med. (2021) 384:1191–203. doi: 10.1056/nejmoa2032125 33789008

[7] Kayani B Okabayashi K Ashrafian H Harling L Rao C Darzi A . Does obesity affect outcomes in patients undergoing esophagectomy for cancer? A meta-analysis. World J Surg. (2012) 36:1785–95. doi: 10.1007/s00268-012-1582-4 22526035

[8] Gale CR Cooper C Sayer AA . Prevalence of frailty and disability: findings from the English Longitudinal Study of Ageing. Age Ageing. (2015) 44:162–5. doi: 10.1093/ageing/afu148 25313241 PMC4311180

[9] Hewitt J Carter B McCarthy K Pearce L Law J Wilson FV . Frailty predicts mortality in all emergency surgical admissions regardless of age. An observational study. Age Ageing. (2019) 48:388–94. doi: 10.1093/ageing/afy217 30778528

[10] Tang A Ahmad U Raja S Rappaport J Raymond DP Sudarshan M . Looking beyond the eyeball test: a novel vitality index to predict recovery after esophagectomy. J Thorac Cardiovasc Surg. (2021) 161:822–32. doi: 10.1016/j.jtcvs.2020.10.122 33451846 PMC8715769

[11] Mayhew D Mendonca V Murthy BVS . A review of ASA physical status - historical perspectives and modern developments. Anaesthesia. (2019) 74:373–9. doi: 10.1111/anae.14569 30648259

[12] Foley C Kendall MC Apruzzese P De Oliveira GS . American Society of Anesthesiologists Physical Status Classification as a reliable predictor of postoperative medical complications and mortality following ambulatory surgery: an analysis of 2,089,830 ACS-NSQIP outpatient cases. BMC Surg. (2021) 21:253. doi: 10.1186/s12893-021-01256-6 34020623 PMC8140433

[13] Shi MK Mei YQ Shi JL . Short-(30-90 days) and mid-term (1-3 years) outcomes and prognostic factors of patients with esophageal cancer undergoing surgical treatments. World J Clin cases. (2022) 10:7708–19. doi: 10.12998/wjcc.v10.i22.7708 36158480 PMC9372832

[14] Gockel I Exner C Junginger T . Morbidity and mortality after esophagectomy for esophageal carcinoma: a risk analysis. World J Surg Oncol. (2005) 3:37. doi: 10.1186/1477-7819-3-37 15969746 PMC1168909

[15] Cesari M Prince M Thiyagarajan JA De Carvalho IA Bernabei R Chan P . Frailty: an emerging public health priority. J Am Med Dir Assoc. (2016) 17:188–92. doi: 10.1016/j.jamda.2015.12.016 26805753

[16] Vermillion SA Hsu F-C Dorrell RD Shen P Clark CJ . Modified frailty index predicts postoperative outcomes in older gastrointestinal cancer patients. J Surg Oncol. (2017) 115:997–1003. doi: 10.1002/jso.24617 28437582 PMC7064809

[17] Hodari A Hammoud ZT Borgi JF Tsiouris A Rubinfeld IS . Assessment of morbidity and mortality after esophagectomy using a modified frailty index. Ann Thorac Surg. (2013) 96:1240–5. doi: 10.1016/j.athoracsur.2013.05.051 23915593

[18] D’Journo XB Boulate D Fourdrain A Loundou A Henegouwen MIB Gisbertz SS . Risk prediction model of 90-day mortality after esophagectomy for cancer. JAMA Surg. (2021) 156:836–45. doi: 10.1001/jamasurg.2021.2376 PMC8223144 34160587

[19] Ko FC . Preoperative frailty evaluation: a promising risk-stratification tool in older adults undergoing general surgery. Clin Ther. (2019) 41:387–99. doi: 10.1016/j.clinthera.2019.01.014 30799232 PMC6585449

