# Supplementary material for: Predictive performance of the Modified Frailty Index and ASA-PS classification for 30-day mortality after esophagectomy in patients with esophageal cancer
Source: Front Oncol. 2026 Jul 22;16:1818115. doi: 10.3389/fonc.2026.1818115 (PMC13437477; doi:10.3389/fonc.2026.1818115)
Supplement: Supplementary file 1 [file Table1.docx]

**Supplementary Table S1.** Distribution of postoperative complications according to the Clavien–Dindo classification

| **Clavien-Dindo grade** | **N (%)** |
| --- | --- |
| Grade 1 | 25 (20.3) |
| Grade 2 | 38 (30.9) |
| Grade 3 | 39 (31.7) |
| Grade 4 | 6 (4.9) |
| Grade 5 | 15 (12.2) |
